# The Use of Humanized Monoclonal Antibodies for the Prevention of Respiratory Syncytial Virus Infection

**DOI:** 10.1155/2013/359683

**Published:** 2013-06-11

**Authors:** Marcello Lanari, Silvia Vandini, Santo Arcuri, Silvia Galletti, Giacomo Faldella

**Affiliations:** ^1^Pediatrics and Neonatology Unit, Imola Hospital, Via Montericco 4, 40026 Imola, Italy; ^2^Neonatology, S. Orsola-Malpighi Hospital, Via Massarenti 11, 40138 Bologna, Italy

## Abstract

Monoclonal antibodies are widely used both in infants and in adults for several indications. Humanized monoclonal antibodies (palivizumab) have been used for many years for the prevention of respiratory syncytial virus infection in pediatric populations (preterm infants, infants with chronic lung disease or congenital heart disease) at high risk of severe and potentially lethal course of the infection. This drug was reported to be safe, well tolerated and effective to decrease the hospitalization rate and mortality in these groups of infants by several clinical trials. In the present paper we report the development and the current use of monoclonal antibodies for prophylaxis against respiratory syncytial virus.

## 1. Introduction

Respiratory syncytial virus (RSV) was discovered in 1956 and it was classified as a member of *Pneumovirus* genus and Paramyxoviridae family. It has an RNA genome which is enveloped, negative-sense single-stranded, and nonsegmented; it is composed of 10 genes encoding for 11 proteins. The envelope is formed by 4 proteins in a lipid bilayer: the two glycosylated surface G and F (fusion) proteins, the M (matrix) protein, and the SH (small hydrophobic) protein.

The G and F proteins play a key role in the pathogenesis of the infection since the G protein determines the adhesion to the cells of the respiratory epithelium, while the F protein is responsible for the entry of the virus in the cells and determines the insertion of viral RNA in the cell which is responsible for the formation of syncytia [1]. 

Two subtypes of RSV, A and B, are different for the G protein structure; RSV A and B coexist during every RSV epidemic season and the subtype A seems to be associated with more severe infections [2, 3]. Neutralizing antibodies against G protein are subtype specific, while antibodies against F protein neutralize both subtypes and could be more useful for active and passive immunization. 

RSV is responsible for respiratory tract infections that could lead to severe respiratory failure and death in infants, especially in those born extremely preterm or affected by some chronic conditions. RSV is a worldwide infection whose specific antibodies are detected in 87% of 18-month-old infants [4] and virtually in all infants older than 3 years. 

RSV is one of the major causes of lower respiratory tract infections (LRTI) during infancy with high rates of hospitalization and mortality during the first years of life [5, 6]. 

The widespread diffusion of the virus and the high interhuman diffusion lead to large epidemics in infants younger than 5 years with a strong economic impact due to the increase of pediatric visits, emergency room accesses, and hospitalizations [7].

Moreover, it was observed that RSV-associated mortality during the first year of life was ninefold higher than influenza-associated mortality [8].

The number of worldwide episodes of RSV infections [9] in children younger than 5 years was estimated to be over 33 million in 2005. During the same year, the number of hospitalizations for severe acute LRTI was estimated to be 3.4 (2.8–4.3) million among young children, with a mortality rate up to 66,000–199,000/year for children <5 years. Ninety-nine percent of all deaths were recorded in developing countries [10], where the disease-specific mortality is nearly 7%. In the developed countries the mortality is far lower (0.5–2%), and severe RSV infections are mostly observed in high-risk infants [4]. 

The incidence of RSV-related hospitalization in the USA increased from 22.2% in 1980 to 47% in 1996 during the first year of life and from 5.4% to 16.4% at any age [11]. The estimated rate of hospitalization in infants younger than 1 year was 92/1000 infants with congenital heart disease (CHD) and 388/1000 infants with chronic lung disease (CLD). According to gestational age (GA), the estimated hospitalization rate was 70/1000 infants born before 28 weeks' GA, 66/1000 infants born at 29–32 weeks' GA, 57/1000 infants born at 33–36 weeks' GA and 30/1000 healthy infants born at term. 

Pediatric patients at high risk of RSV morbidity and mortality include preterm infants, especially those with CLD, infants with CHD, neuromuscular diseases, cystic fibrosis, and congenital or acquired immunodeficiency [12, 13].

Considering the great impact of RSV infections on children health, several studies were conducted to identify risk factors and to develop an effective tool for specific prophylaxis in high-risk infants.

Prematurity is one of most important risk factors for severe RSV infections in young infants because of their immature immune response and their incomplete development of the lungs and the airways. Low GA is one of the most relevant risk factors for RSV-related hospitalization during the first months of life: the yearly hospitalization rate is lower in term infants (4.4%) than in infants born before 28 weeks' GA (9.4%) [14].

Multicenter studies were conducted to analyze RSV epidemiology in large cohorts of term and preterm infants; a higher risk for RSV infections was reported among preterm infants, including also late preterm infants (33–35 weeks GA); these studies also evaluated the risk factors for RSV-related infection and hospitalization in preterm infants [15–22]. The knowledge of the epidemiology of RSV infection and its risk factors is extremely useful to improve the use of the prophylaxis with palivizumab.

A multicenter study conducted in Italy [17] reported a higher rate of RSV infections in infants with GA ≤ 35 weeks than in infants born at term.

Risk factors for severe RSV infections in late preterm were also investigated in the PICNIC study (Pediatric Investigators Collaborative Network on Infections in Canada) by Wang et al. [15] (1995) and in the FLIP study [16] which investigated risk factors that most likely may lead to development of RSV-related respiratory infection and subsequent hospital admission among premature infants born 33–35 weeks' GA.

Moreover, a multicenter cohort Italian study started in November 2009 enrolled newborns born at GA ≥ 33 weeks: preliminary data showed that infants of the lower GA group (33 weeks + 0 days–34 weeks + 6 days) were at a slight higher risk of hospitalization for LRTI during the first year of life [18].

Risk factors for RSV hospitalization reported in these three studies are summarized in Table 1.

CLD is a chronic pulmonary disease which may affect premature infants characterized by oxygen requirement after 28 days of age [23]. The pathogenesis of CLD is multifactorial and is related to prenatal (chorioamnionitis, intrauterine growth restriction) and postnatal (ventilator-induced lung injury, oxidative stress, infections, steroids, pulmonary fluids overload, and nutritional deficits) factors which interfere with lung development before and after preterm birth.

The RSV-related hospitalization rate in infants younger than 6 months with CLD is 56.2/100 children/year [5]. The severity of RSV infections in this population is related to the reduction of lung volume and the airways hyperreactivity, deformation, and inflammation. 

In our experience [24], infants with CHD waiting for surgical repair are at high risk of nosocomial RSV infections (9.8%).

Other congenital malformations are associated to a more severe course of RSV LRTI; in a birth cohort study conducted in Colorado from 1997 to 2004, the risk for RSV-related hospitalization was higher in infants <2 years with spina bifida, agenesis, hypoplasia or dysplasia of the lung, cleft palate alone, and biliary atresia [25].

Congenital or acquired immunodeficiency may also increase the risk of RSV related hospitalization.

Moreover, Luján-Zilbermann et al. [26] reported that the risk for severe respiratory virus infections increased after hematopoietic stem cells transplantation; 14% of the infections detected in this cohort were determined by RSV.

Neuromuscular diseases are also associated with severe respiratory infections, especially in the presence of technology dependence and respiratory support [27]; in a prospective multicenter study conducted in Germany during 6 consecutive RSV seasons (1999–2005), 4.7% of infants with infection had a clinically relevant neuromuscular disease associated to increased risk for seizures (15.1% versus 1.6%), need of mechanical ventilation (9.6% versus 1.9%), and death (5.5% versus 0.2%) [28]. These data were confirmed by Resch et al. [13], who reported that RSV LRTIs are frequently more severe in infants with neuromuscular diseases, because of low pulmonary capacity, coexisting gastrooesophageal reflux and muscle weakness; these conditions lead to impaired cough reflex with increased risk of aspiration and atelectasis. 

The prevention of RSV infections in infants is extremely important to decrease the great amount of complications and hospitalization in young infants. For this reason, pharmacological research during the last 20 years aimed at the development of a safe, well-tolerated, and effective drug and led to the current use of monoclonal antibodies.

## 2. The “History” of the RSV Prophylaxis 

The development of a safe, effective, and well-tolerated drug for RSV prophylaxis in high-risk infants has been studied for many years through clinical trials. Many authors reviewed the “history” of the development of the drug used for specific prophylaxis [29–32].

Passive prophylaxis was at first introduced in the 1990s with standard intravenous immunoglobulin (IGIV) after studies developed in cotton rats [33]; neutralizing antibody titer was detected, and it determined virus reduction mainly in the lower airways. After these results in animal models, clinical trials based on the administration of IGIV to high-risk infants monthly during the RSV season were started [34, 35] and reported no statistically significant decrease in the severity of RSV infection, no major adverse events, and a slight decrease in the length of hospital stay. This lack of efficacy could be explained by the insufficient anti-RSV antibody concentration in standard immunoglobulin; for these reasons, the evaluation of the efficacy of a hyperimmune RSV polyclonal globulin (RSV-IVIG) was the subject of two multicenter randomized controlled trials [36, 37]. These studies evaluated the response to five doses of RSV-IGIV administered monthly to preterm infants during the epidemic season and reported a 41–63% decrease in hospital admissions. The use of this drug was invalidated by some drawbacks [38]: the need of an intravenous access, large fluid infusion (15 mL/kg), supply shortages, high protein load (750 mg/kg), theoretical risk of transmission of blood-borne infections, and possible interference with pediatric vaccines.

The effectiveness of RSV-IGIV to prevent RSV infections requiring hospitalization in children with cardiovascular disease was analyzed in a randomized controlled trial enrolling 416 children younger than 4 years with CHD or cardiomyopathy [39]. Monthly RSV-IGIV infusion (750 mg/kg) during the RSV season did not reduce the number of RSV-related hospitalizations, even if it slightly reduced the number of hospital admissions for any respiratory tract infection. The clinical trials investigating the safety and effectiveness of RSV-IGIV were interrupted because of the high incidence of sudden cyanotic adverse events and the worsening of the outcome following cardiac surgery [39, 40]. The use of RSV-IGIV was completely withdrawn in 2003.

### 2.1. The Use of Monoclonal Antibodies for RSV Prophylaxis

Beyond the environmental prevention used to limit the virus diffusion especially in hospitalized patients, nowadays the prophylaxis of RSV-related hospital admissions is based on the administration of specific monoclonal antibodies to the infants at high risk.

At first, a murine monoclonal anti-RSV antibody (mAb) was developed; it was an IgA intended for topical nasal administration [41]. However, the clinical study did not overcome phase III.

The mAbs neutralizing G surface glycoprotein are not enough effective because of the variability of this protein between the two viral subtypes A and B. F protein has less heterogeneity, and it is stable in different seasons and in different geographic areas [42]; thus it has become the ideal target for the two specific mAbs: SB 209763 and palivizumab.

SB 209763 is an IgG1 specific antibody against the C epitope of the F protein. Its effectiveness is unclear because a large, multicenter, placebo-controlled clinical trial did not report a significant reduction in the number of RSV-related hospital admission after the monthly 10 mg/kg dose administered to 800 European and American children [43].

Palivizumab is an IgG1 antibody specific for a different epitope (A) of F protein that was introduced in the United States in 1998; it is currently the only approved monoclonal antibody used for RSV prophylaxis [44, 45]. It is composed of two sequences, a human one (95%) and a murine one (5%).

The mAbs have the same properties of a human IgG1, with a long half-life (28 days). It is free from the risk of transmission of blood-borne pathogens and it can be produced in large batch lots that provide sufficient supply. 

Beeler and Van Wyke Coelingh in 1989 [44] evaluated in a murine model the biological properties of the different epitopes of the RSV F glycoprotein and reported that sites A and C were involved in viral fusion activity. Epitope A and epitope C were reported to be less variable than epitope B and for this reason their neutralizing activity against glycoprotein F is more stable. Moreover, epitope A appeared to be involved in the viral fusion which leads to the formation of syncytia; subsequently, mAbs-neutralizing epitope A contrasts syncytia formation. 

Palivizumab reduces RSV replication [46] through the inhibition of the virus fusion with the lung endothelial cells; it has no effect on viral attachment and interaction with target cells and it does not reduce the viral budding.

The biological characteristics of palivizumab were analyzed in cotton rat models [47]. The rats received an injection of antibodies and on the following day they received RSV subtype A and B intranasally. The reduction of the replication of both viral subtypes was greater than 99% after an IV dose of 2.5 mg/kg, obtaining a titer of serum antibodies near 25–30 *μ*g/mL. This serum concentration was considered to be a protective value for human receiving palivizumab to prevent RSV infections.

Several trials were also conducted to determine if a noninhibitory concentration of antibodies could promote viral replication or virus-mediated respiratory disorders; this hypothesis was derived from previous studies conducted in the 1960s [48]. It was reported that the animal lung tissue grew a single viral plaque after the administration of a very low dose of palivizumab (0.0032 mg/kg), and the immunized animals were completely resistant to infection after the clearance of the drug [47]. 

The possibility of failure of palivizumab efficacy due to genetic variation of the A epitope of the F protein was excluded by surveillance studies [49, 50].

The pharmacokinetic characteristics of palivizumab after a single intramuscular (IM) 15 mg/kg injection were analyzed with the aim of defining the prophylaxis schedule [51, 52]; the mean half-life was 20–30 days with highly variable serum concentration 30 days after each dose. 

The effect of the drug on RSV detection in the airways was investigated in hospitalized children with severe RSV disease after an intravenous (IV) dose [53]. The RSV antigen concentration decreased only in the lower respiratory airways, confirming that the reduction of virus concentration by 99% in the nasopharynx requires an antibody titer 10-fold higher than the neutralizing titer needed in the lungs [54].

A study conducted to investigate pharmacokinetic properties of palivizumab in infants <2 years without CHD receiving 15 mg/kg of palivizumab IM monthly reported a half-life of approximately 20 days and a mean serum antibody titer (mean ± standard error, SE) of 37 ± 1.2 *μ*g/mL after the first dose, 57 ± 2.4 *μ*g/mL after the second dose, 68  ±  2.9 *μ*g/mL after the third dose, and 72 ± 1.7 *μ*g/mL after the fourth dose. Serum antibody concentration in infant with CHD was not different after the first and the fourth doses [51]. 

Children who received palivizumab in the previous epidemic season had mean concentrations 60.7 ± 2.4 *μ*g/mL after the first administration and 86.2 ± 4.2 *μ*g/mL after the fourth administration in the second season of prophylaxis [55].

Another study administered palivizumab with the same schedule to 139 infants younger than 2 years with haemodynamically significant CHD and reported a serum concentration of 98 ± 52 *μ*g/mL before cardiac bypass and of 41 ± 33 *μ*g/mL after bypass; this 58% decrease had no defined clinical consequences [56]. Pharmacokinetic characteristics of palivizumab were also determined in a cohort of Japanese adults [57]. A phase I safety trial was conducted in six Japanese and six overseas adults who received 3 mg/kg IM, 3 mg/kg IV, 10 mg/kg IV, and 15 mg/kg IV of palivizumab. The IV infusion was preferred because of the large volume of fluid in the dose for adult patients; the pharmacokinetic properties were similar for both IM and IV administrations [58]. Maximum concentrations, area under the curve (AUC), half-life, and clearance were similar in the two groups of adult volunteers; no adverse events were registered. 

Subsequently a phase II trial was conducted enrolling 31 Japanese children (19 preterm and 13 with CLD) with the previous prophylaxis schedule; mean serum titer through levels and AUC was not different in both Japanese and overseas infants [57]. No adverse events occurred; a case of mild RSV upper respiratory tract infection (URTI) not requiring hospitalization was reported. 

These pharmacokinetics trials guided the development of current indications for palivizumab use.

The monthly schedule of palivizumab administration should be continued also if an RSV infection occurs; the prophylaxis should start before the beginning of the RSV season (from November to April in the northern hemisphere) [59]. 

Since the antibody serum titer is lower after cardiac bypass, patients undergoing this procedure should receive an injection of palivizumab as soon as possible after surgery.

The monthly dose should be injected by aseptic technique in the anterolateral area of the thigh, avoiding the gluteal area because of the risk of injury of the sciatic nerve. Volumes over 1 mL should be administered in divided doses. 

The efficacy and safety of palivizumab were investigated in two randomized, double-blind, placebo-controlled trials: the Impact-RSV trial [55] and another study conducted in children with haemodynamically significant CHD [56].

The Impact trial was performed during one RSV season in 1998 and enrolled a cohort of 1502 infants younger than 2 years with CLD or infants born before 36 weeks' GA and aged less than 6 months. The cohort was divided as follows: 1002 randomized children received 15 mg/kg palivizumab at monthly intervals, and 500 received placebo during the RSV season; all children were followedup for 150 days after the enrollment. Primary endpoints were an RSV-positive respiratory illness requiring hospital admission or a moderate respiratory illness in infants who had had a positive RSV test during a previous hospital stay; the number of RSV-related hospitalizations decreased by 55% in the overall palivizumab group, with a reduction in days of hospitalization; the number of days requiring mechanical ventilation, days of hospitalization for other causes, and the incidence of otitis media did not differ between the two groups. This reduction was greater in preterm infants (78%; 95% CI 66–90%) than in infants with CLD (39%; 95% CI 20–58%), who often require hospitalization also for mild respiratory disease. The reported adverse events were not different between the two groups and included injection site reactions (2.7% in palivizumab group versus 1.8% in placebo group), rash (0.9% versus 0.2%), fever (2.8% versus 3%), and nervousness (2.6% versus 2.5%). The mortality rate was 0.4% in the palivizumab group and 1% in the placebo group; none of the deaths was related to palivizumab administration.

The mortality during RSV-related hospitalization was 6.7% in studies that included deaths for causes not related to the infection [15, 20].

The efficacy and safety of palivizumab in two seasons were analyzed in 88 infants of the Impact study [55]. The prevalence of anti-palivizumab antibodies (titer > 1/40) was observed in one subject and did not determine serious adverse events.

A randomized, double-blind, placebo-controlled multicenter trial enrolled 1287 children aged ≤2 years with haemodynamically significant CHD [56] who received monthly 15 mg/kg doses of palivizumab for five months. This trial reported a reduction of the incidence of hospital admissions (5.3% versus 9.7%), length of hospitalization for RSV infection, and total hospital days with requiring of supplemental oxygen. The study cohort included both cyanotic and noncyanotic CHD; the reduction of hospital admission was 29% in the cyanotic group and 58% in the noncyanotic group. 

The incidence of adverse events (fever, injection site reactions, conjunctivitis, and cyanosis) was not different in the palivizumab group and in the placebo group and never led to drug discontinuation. Mortality was not different in the two groups and no deaths were related to palivizumab; moreover, the drug did not affect the management of the CHD.

A recent review [60] stated that prophylaxis with palivizumab is effective to prevent hospitalization for RSV bronchiolitis in infants with CHD, since an effective vaccine is not yet available. Pharmacoeconomic studies were also reviewed; the cost-effectiveness of palivizumab's use in infants with CHD was confirmed. 

Palivizumab was also reported to reduce by 80% the risk of recurrent wheezing in children aged 2–5 years and born prematurely, with a 68% decrease in infants without family history of asthma and 80% in infants without atopic background, but this protective effect was not observed in infants with an atopic family [61]. This result could be explained by the fact that RSV-related recurrent wheezing is not mediated by an atopic response. 

Moreover, a prospective Italian study comparing 154 palivizumab recipients to 71 palivizumab nonrecipients [62] observed a decrease in hospital admissions after palivizumab administration in infants <6 months at the beginning of their first RSV season. 

The treatment of bronchiolitis is essentially based on respiratory support, adequate fluids and nutrition supply, and therapy of respiratory symptoms, since an etiologic therapy does not exist. The safety and effectiveness of palivizumab in children hospitalized for acute RSV infections were investigated in a phase I/II, randomized, multicenter, placebo-controlled, double-blind trial that reported the absence of differences in clinical outcomes between palivizumab and placebo group; no adverse events requiring trial interruption were observed [63]. 

Another drug of the group of monoclonal antibodies (motavizumab, Medi-524 MedImmune) is a molecule that differs from palivizumab in 13 amino acid residuals; both mAbs are specific for the site A of the F protein of respiratory syncytial virus. The neutralizing effect was measured both in vitro and in animal models and found to be higher for motavizumab [64–66].

Large trials examined clinical effects of this drug for RSV prophylaxis. A double-blind, randomized, phase 2 trial [67] compared properties of palivizumab and motavizumab injected sequentially to 260 infants younger than 2 years with major risk factors for severe RSV infections who received 15 mg/kg palivizumab or motavizumab IM for 5 doses at monthly intervals. Adverse events had the same incidence in all the study groups; mortality was not related to the drug administration. Mean drug serum titer did not differ in the study groups.

A multicenter phase 3 trial involving more than 300 centers [68] analyzed the safety and efficacy of motavizumab in 6635 preterm infants younger than 6 months or younger than 24 months with CLD. The infants received 5 monthly 15 mg/kg doses of palivizumab or motavizumab and were involved in a 5 months followup. Motavizumab was reported not to be inferior to palivizumab in the decrease of RSV-related hospital admissions and RSV LRTI in nonhospitalized patients; the reported adverse events were comparable for the two antibodies. 

Safety, immunogenicity, and pharmacokinetics of motavizumab were determined in a randomized, open-label phase I-II study enrolling 217 high-risk children; motavizumab characteristics were comparable to palivizumab [69].

In January 2008 MedImmune submitted to the Food and Drug Administration the request of a biologic license application for motavizumab, but the advisory committee refused to endorse the request for the use of motavizumab in the prophylaxis of RSV infections in the high-risk pediatric population because it had the same safety, efficacy, and tolerability of palivizumab, which has been in use since the 1990s.

Since palivizumab is a safe, well-tolerated, effective but expensive drug, its use is regulated by national and international guidelines based on pharmacoeconomic studies.

The use of the prophylaxis also in late preterm infants has been a matter of concern for many years [70, 71], since this group is at risk of frequent respiratory infections for the immaturity of the respiratory and the immune system; however, the American Academy of Pediatrics guidelines recommend the use of palivizumab only in the first three months of life, in the presence of risk factors for severe disease, in infants born at 32–34 weeks and 6 days, on daycare attendance, and with 1 or more siblings or other infants <5 years old living in the same house. 

The prophylaxis was observed to be useful especially in late preterm infants with >1 risk factors for RSV LRTI (birth weight, chronological age, presence and age of siblings, and daycare attendance) [72]. 

A prospective, multicenter Italian study [17] including 1232 infants in the first 2 years of life confirmed these data and reported in addition an increase of risk for severe RSV LRTI associated with tobacco smoke exposure.

Clinical trials are ongoing in order to establish the cost-effectiveness of prophylaxis in this population and to improve the existing guidelines for prophylaxis schedule with the support of epidemiological data. 

A cost-utility analysis was made in 4 cohorts of preterm infants [73]; palivizumab was observed to reduce costs and improve QALY (quality-adjusted life year) in preterm infants <32 weeks. Moreover, it was demonstrated to be cost-effective in infants with 32–34 weeks' GA with the risk factors described by the American Academy of Pediatrics in 2009 and in infants with 32–35 weeks' GA with 2 or more risk factors of the report published in 2006.

The positive impact of prophylaxis with palivizumab on healthcare expense was also confirmed by two retrospective analyses made in Austria [74] and in Spain [75].

A pharmacoeconomic study could also improve the guidelines in use for the prophylaxis of RSV infections in infants with other risk factors than prematurity [76]. 

Palivizumab cost-effectiveness was confirmed in infants with neuromuscular diseases, congenital diaphragmatic hernia, but it is still controversial in infants with other diseases (Down syndrome, cystic fibrosis).

## 3. Conclusions

RSV has a worldwide diffusion and it is responsible for a large amount of hospital admissions in young infants, with a subsequent strong impact on infants' health and healthcare costs. RSV infections may lead to respiratory failure and death, especially in infants with chronic pathological conditions. An etiologic treatment for RSV bronchiolitis does not exist at the present, while the use of palivizumab for prophylaxis of RSV infections in high-risk infants is recommended since it is well tolerated and effective in the reduction of RSV-related hospitalizations. Palivizumab is the only monoclonal antibody currently used for anti-infectious purpose.

The extension of prophylaxis with palivizumab to late preterm infants (GA 33–35 weeks) is still a matter of concern since this drug is too expensive to be used for the entire population of late preterm infants. The cost-effectiveness of the use of palivizumab in the late preterm has been analyzed by several studies to identify environmental or individual risk factors for severe RSV infection. The use of risk scores derived from this study is helpful to detect the subjects for whom the administration of palivizumab could be effective to reduce RSV-related mortality and morbidity.

These positive results subsequent to the use of palivizumab to prevent an infectious disease could encourage the use of monoclonal antibodies for prevention and treatment of other infectious diseases in infants and adults. 

## Figures and Tables

**Table 1:** 

Study	Study design	Population	Risk factors
PICNIC	Prospective	Infants hospitalized for RSV infections	(i) Underlying disease (CHD, CLD, immunodeficiency, multiple congenital malformations). (ii) Postnatal age <6 weeks. (iii) GA 33–35 weeks.

FLIP	Prospective case-control study	Preterm born at 33–35 weeks' GA (risk factors for RSV-related hospitalization)	(i) Chronologic age ≤ 10 weeks at the beginning of RSV epidemic season. (ii) Breastfeeding ≤ 2 months. (iii) ≥1 school-age siblings. (iv) ≥4 residents or visitors at home. (v) Family history of wheezing.

Multicenter Italian birth cohort	Multicenter prospective cohort study	Infants born at 33 weeks' GA or more	(i) GA 33 + 0–37 + 6 weeks (ii) No breastfeeding. (iii) Presence of siblings. (iv) Maternal smoking. (v) Family history of atopy or wheezing.

## Abbreviations

RSV: Respiratory syncytial virus
CHD: Congenital heart disease
CLD: Chronic lung disease
LRTI: Lower respiratory tract infection
GA: Gestational age
IVIG: Intravenous immunoglobulin
mAbs: Monoclonal antibodies
IV: Intravenous
IM: Intramuscular
SE: Standard error
AUC: Area under the curve
URTI: Upper respiratory tract infection
QALY: Quality-adjusted life year.
